# Different Association of Human Papillomavirus 16 Variants with Early and Late Presentation of Cervical Cancer

**DOI:** 10.1371/journal.pone.0169315

**Published:** 2016-12-30

**Authors:** Ana Alfaro, Eligia Juárez-Torres, Ingrid Medina-Martínez, Norma Mateos-Guerrero, Maura Bautista-Huerta, Edgar Román-Bassaure, Nicolás Villegas-Sepúlveda, Jaime Berumen

**Affiliations:** 1 Unidad de Medicina Genómica, Facultad de Medicina, Universidad Nacional Autónoma de México / Hospital General de México, México City, México; 2 Servicio de Oncología, Hospital General de México, México City, México; 3 Departamento de Biomedicina Molecular, Centro de Investigación y Estudios Avanzados del Instituto Politécnico Nacional, México City, México; 4 Departamento de Medicina Experimental, Facultad de Medicina, Universidad Nacional Autónoma de México, México City, México; Albert Einstein College of Medicine, UNITED STATES

## Abstract

The median age of cervical cancer (CC) presentation coincides with the mean age of menopause presentation (49 years) in Mexico. Here, we investigated the association between different HPV16 variants and early (≤ 49 years) or delayed (≥ 50 years) CC presentation. We conducted a case-case study that included 462 CCs, 386 squamous cell carcinomas (SCC), 63 adenocarcinomas (ACC), and 13 additional cell types. Variants were identified by PCR and DNA sequencing. The risk conferred by each variant for developing CC earlier than 50 years was analyzed using a univariate logistic regression model considering old-aged patients (≥ 50 years) and non-HPV16 cases as the reference variables. Overall, the frequency of HPV16 was 50.9%, and the only identified variants were the European A1/2 (31.2%) and the Asian-American D2 (10.8%), and D3 (8.9%). D2 was mainly associated with ≤ 49-year-old patients (15.9%); A1/2 was uniformly distributed between the two age groups (~31%), whereas D3 increased with age to a frequency of 11.8% in the older group. Only the D2 variant conferred a 3.3-fold increase in the risk of developing CC before 50 years of age (OR = 3.3, 95% CI = 1.7–6.6, p < 0.001) in relation with non-HPV16 cases. Remarkably, this risk was higher for ACC (OR = 6.0, 95% CI = 1.1–33, p < 0.05) than for SCC (OR = 2.8, 95% CI = 1.3–5.9, p < 0.01). Interestingly, when analyzing only the HPV16-positive CC, D2 increases (OR = 2.5, 95% CI = 1.2–5, p < 0.05) and D3 decreases (OR = 0.45, 95% CI 0.2–0.9, p < 0.05) the risk to develop CC before 50 years old in relation with A1/2 variant. These results indicated that D2 variant is associated with early and D3 with delayed CC presentation, whereas A1/2 variant was uniformly distributed between the two age groups.

## Introduction

Cervical cancer (CC) is the fourth most frequent cancer in women worldwide; over 500,000 new cases are identified each year, and it is the fourth cause of cancer-related death among women in developing countries [1]. Human papillomavirus (HPV) is the main factor affecting the development of CC [2, 3]. HPV16 is the most common viral type worldwide and is found in approximately 50% of CC cases, followed by HPV18, HPV45, and HPV31 [3]. The incidence of HPV in healthy women varies with age [4]. In most studies, HPV occurrence peaks in young women (<25 years), and then the incidence decreases with age. In contrast, CC distribution follows a standard curve, peaking at approximately 50 years of age. Therefore, half of CC cases are diagnosed in young premenopausal women and half in postmenopausal women [5, 6]. Most CC cases arise at 15–20 years after initial HPV infection [7]. According to the frequency distribution of HPV in healthy women, this latency may explain most CC cases in young and middle-aged women, but not the cases in women over 50 years of age [8]. These women could have acquired HPV shortly before the disease presentation or many years prior. In any case, these data suggest that the events leading to cervical carcinogenesis in elderly patients may differ from those in younger patients.

The mean age of patients with CC who are positive for HPV16, 18 or 45 is lower than that of patients infected with other HPVs [9, 10]. In a previous study in Mexico, we found that the percent positivity of high-risk HPVs in CC cases varies with the age of the patients [9]. Three different trends were identified, one each for HPV16, HPV18/45, and other HPVs. In the case of HPV16, the percent positivity peaked (63.2%) at ages ≤ 35 years of age; and then gradually decreased until 56–60 years of age (31.1%). A second peak (52.5%) was found at 61–65 years, which was followed by a decrease with age. The percent positivity of HPV18/45 showed a decreasing trend from younger (19.3%) to older (>70 years, 12.8%) women. In contrast, the percent positivity of the remaining high-risk HPVs increased from younger (15.8%) to older (46.2%) women. These data indicate that most (>80%) CCs in young women depend on the presence of highly oncogenic HPVs (16, 18, and 45 types) [11–14]. In contrast, nearly half of the CC cases in older patients were associated with less oncogenic high-risk HPVs.

The bimodal trend of HPV16 positivity in CC by age [9] suggests the presence of two different types of infection due to different viral variants, genetic or physiological changes, or differences in patient lifestyle factors. Previous findings support the hypothesis of HPV16 variants. In Mexico, it has been shown that nearly 40% of infections by HPV16 are due to Asian–American lineage variants, D2 (AA-c) and D3 (AA-a), which confer a nine-fold greater risk than European lineage variants A1/2 (E) for the development of CC. In addition, D2-positive patients were, on average, 6 years younger than patients positive for A1/2 and D3 [15]. These data suggest that D2 could be more aggressive and associated with younger patients, and other HPV16 variants could be associated with older patients in Mexico. To investigate whether different HPV16 variants are associated with early or delayed presentation of CC, we conducted a case-case study. The cases of a previously published study [9] were used for the analysis of HPV16 variants.

## Materials and Methods

### Patient selection and study design

The cases of a previously published cross-sectional study were used for this analysis [9]. Five hundred and three patients newly diagnosed with CC (incident cases) were recruited at the Oncology Department of the Hospital General de Mexico in Mexico City, which treats patients without Social Security in the metropolitan area. In fact, two thirds of patients recruited in the present study occasionally or never have attended a screening program. Inclusion criteria were clinical diagnosis of invasive CC at the Oncology Department, no previous treatment, born and residing in Mexico, and Mexican ancestry of at least two generations. Patients fulfilling the inclusion criteria were sequentially recruited from November 2003 to April 2005 and from January 2006 to July 2007 and represented approximately 80% of the patients diagnosed with CC during this period.

All subjects received a complete clinical evaluation by an experienced oncologist. Tumor staging was carried out according to the International Federation of Gynecology and Obstetrics (FIGO) [16]. Forty-one patients were excluded because of poor-quality biological samples or because they were confirmed to have high-grade squamous intraepithelial lesions instead of CC by three pathologists. After exclusion, 462 CC patients remained in the study group. The participation rate of case subjects was 95% [15, 17].

For HPV detection and typing, cervix scrapes were collected using a cytobrush on the same day the patients were recruited. Cells were suspended in a vial containing an extraction buffer (10 mM Tris-HCl pH 7.6, 5 mM EDTA, 150 mM NaCl, 1% SDS) and stored at −20°C until analysis. The percent positivity of viral variants was compared between early (≤ 49 years of age; n = 233) and late (≥ 50 years of age; n = 229) CC presentation. The risk conferred by HPV16 variants for the development of CC before 50 years of age was calculated by considering the old-aged patients as reference of the outcome variable. The study protocol was approved by the Scientific and Ethics Committees of the Hospital General de Mexico (approval number DIC/03/311/04/051), and informed written consents were obtained from all participants prior to their inclusion.

### Detection and HPV typing of HPV16 variants

The frequency of HPV types, which were detected by PCR with universal primers, was previously published [9]. From the total HPV16-positive samples (n = 235) included in this analysis, 90.6% (n = 213) had a single HPV16 infection and only 9.4% (n = 22) had an additional HPV type (double infection). HPV16 variants were detected in case specimens by polymerase chain reaction, as previously described [15, 18], using suitable primers for *E6/E7* (forward 5′-ATGCACCAAAAGAGACTGC-3′, position 083, reverse 5′-TTATTGTTTCTGAGAACAGA-3′, position 858), the MY region of the *L1* gene (forward 5′-GCACAGGGCCACAATAATGG-3′, position 6584, reverse 5′-CGTCCTAAAGGAAACTGATC-3′, position 7035) and the LCR region (forward 5’-TATTTTGGAGGACTGGAATTTT-3’, position 6829, reverse 5’-TCTGTGCATAACTGTGGTAACTTTCTG-3’, position 156). HPV16 variants were identified by sequencing *E6*, MY, and LCR regions, as previously described [15]. Sequences were analyzed using the FASTA sequence similarity tool [19, 20], the SeqScape software (Applied Biosystems, Foster City, CA, USA), and the ClustalW2 alignment tool (http://www.ebi.ac.uk/Tools/msa/clustalw2/). HPV16 sequences and base positions were aligned with reference sequences of specific sublineages as follows: NC_001526.4 [reference sequence (7906 pb), Sublineage A1], AF536179 (Sublineage A2), AY686579 (Sublineage D2) and AF402678 (Sublineage D3). Identification of the HPV16 lineages and sublineages was based on single nucleotide variants (SNVs) of positions proposed by Yamada et al. [21] and Burk et al. [22]. In addition, for D variant classification we specially used the diagnostic SNPs in the LCR, positions 7507 and 7743 [23], and the MY, positions 6803 and 6862 [21]. The reproducibility of the HPV16 testing was 99.2% and that of the variant analysis was 97.6%.

### Statistical analysis

Patient age results were expressed as the median and interquartile range (IQR = 25–75%), and the Mann-Whitney U test was performed to assess the statistical significance of differences among the groups. A comparison of percent positivity of HPV16 variants was made among patients with early and late CC presentation. The significance of the differences among the groups was assessed by Pearson chi-square test or Fisher exact test. The percent positivity of HPV16 variants was also analyzed considering 5-year intervals and the resulting trends were analyzed by the Spearman correlation. The risk conferred by HPV16 variants (explanatory variable) was calculated using a univariate logistic regression model (LRM). When all samples were included in the analysis, the non-HPV16 cases were considered the reference of the explanatory variable. Non-HPV16 cases included HPV-negative samples (n = 7) and samples positive for HPVs other than HPV16 (n = 220). When only the HPV16-positive samples were analyzed, the A1/2-positive infection was considered the reference of the explanatory variable. The association was expressed as the odds ratio (OR) and the 95% confidence interval (CI). All statistical tests were two-sided; differences were considered significant when p < 0.05. The statistical analyses were conducted using Sigma Plot (Systat Software, Inc., San Jose, CA, USA) or SPSS ver. 20 software (SPSS, Inc., Chicago, IL, USA).

## Results

### Frequency and identification of HPV16 variants in CC

The HPV16 percent positivity in CC was 50.9% (235/462), including only European sublineages A1/2 (31.4%, 144/462), and Asian–American sublineages D2 (10.4%, 50/462) and D3 (9.1%, 41/462; Table 1). Therefore, AA variants represent 38.7% (91/235) of HPV16-positive cases (Table 1). These rates were similar to those previously reported in CC [15]. We did not find any sample positive for more than one HPV16 variant, and the distribution of HPV16 variants was not different between CC positive for single HPV16 and those positive for HPV16 and another HPV type (S1 Table).

**Table 1:** Association between HPV16 variants and early presentation of cervical cancer.

HPV16 variants	Age Group: % (n)			OR (95% CI)	p value
	≤49	≥50^a^	Total		
**All cases**					
HPV16 negative^b^*	45.1 (105)	53.3 (122)	49.1 (227)	1	
HPV16 positive	54.9 (128)	46.7 (107)	50.9 (235)	1.4 (1–2)	0.078
A1/A2	33.0 (77)	29.3 (67)	31.2 (144)	1.3 (0.9–2)	0.176
D2	15.9 (37)	5.7 (13)	10.8 (50)	**3.3 (1.7–6.6)**	**0.001**
D3	6.0 (14)	11.8 (27)	8.9 (41)	0.6 (0.3–1.2)	0.154
Total	100 (233)	100 (229)	100 (462)		
**Squamous cell carcinoma**					
HPV16 negative^b^*	46.6 (90)	54.4 (105)	50.5 (195)	1	
HPV16 positive	53.4 (103)	45.6 (88)	49.5 (191)	1.4 (0.9–2)	0.127
A1/A2	35.2 (68)	30.6 (59)	32.9 (127)	1.3 (0.9–2.1)	0.195
D2	13.5 (26)	5.7 (11)	9.6 (37)	**2.8 (1.3–5.9)**	**0.009**
D3	4.7 (9)	9.3 (18)	7 (27)	0.6 (0.2–1.4)	0.213
Total	100 (193)	100 (193)	100 (386)		
**Adenocarcinoma**					
HPV16 negative^b^*	37.5 (12)	51.6 (16)	44.4 (28)	1	
HPV16 positive	62.5 (20)	48.4 (15)	55.6 (35)	1.8 (0.7–4.9)	0.261
A1/A2	18.8 (6)	19.4 (6)	19 (12)	1.3 (0.3–5.2)	0.678
D2	28.1 (9)	6.5 (2)	17.5 (11)	**6 (1.1–33)**	**0.039**
D3	15.6 (5)	22.6 (7)	19 (12)	1 (0.2–3.7)	0.944
Total	100 (32)	100 (31)	100 (63)		
**HPV16-positive cases**^c^					
A1/A2*	60.2 (77)	62.6 (67)	61.3 (144)	1	
D2	28.9 (37)	12.1 (13)	21.3 (50)	**2.5 (1.2–5.0)**	**0.013**
D3	10.9 (14)	25.2 (27)	17.4 (41)	**0.45 (0.2–0.9)**	**0.031**
Total	100 (128)	100 (107)	100 (235)		

Odds ratios were calculated using a logistic regression model.

Reference variable * (OR = 1), p value and 95% confidential interval are shown.

a. ≥50-year-old CC group was taken as reference group.

b. HPV16 negative included HPV-negative samples and samples positive for HPVs other than HPV16.

c. ORs were not statistically significant when these cases were stratified by histology (S3 Table).

**Fig 1:**
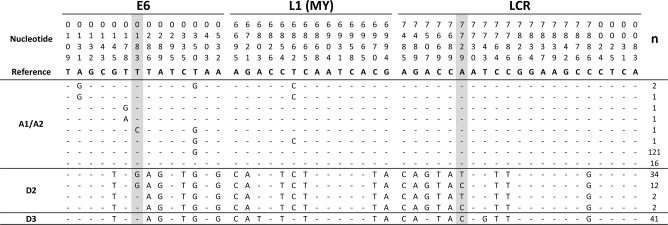
Nucleotide sequence changes in E6, L1 (MY), and LCR regions from A1/2, D2 and D3 HPV16 variants. The figure shows nucleotide changes in the E6, L1 (MY) and LCR regions which were amplified from 235 DNA isolates derived from cervical carcinomas positive for HPV16. The nucleotide positions at which variations were observed are written vertically. Classification of HPV16 variants were performed according to Yamada et al. [21] and Burk et al. [22]. The E6 panel shows 14 variant positions, all used by Yamada and all but one (183) by Burk. The LCR panel shows 21 positions, all used by Burk and Yamada. The MY panel shows 14 positions that were used by Yamada. Variant-sequence positions that do not vary in relation with the HPV16-reference sequence are marked with dashes. The number of positive CC samples (n) is shown at the right side. The HPV16R sequence (7906 bp), listed as NC_001526.4 in GenBank, was used as the reference sequence for all the alignments. The reference sequences used to classify the specific sublineages were as follows: NC_001526.4 (Sublineage A1), AF536179 (Sublineage A2), AY686579 (Sublineage D2) and AF402678 (Sublineage D3). In addition, for D variant classification we specially used the diagnostic SNPs in the LCR, positions 7507 and 7743 [23], and the MY, positions 6803 and 6862 [21].

The identification of sublineages was initially done according to the method reported by Yamada et al. [21], using the positions in the *E6* and MY regions (Fig 1). Subsequently, we confirmed these findings by analyzing the majority of the positions in E6 (n = 13) and LCR (n = 21) proposed by Burk et al. [22] for the classification of HPV16 variants. However, we found some interesting novelties in E6 and LCR among the AA lineages. All 13 positions in E6 of the AA lineages in our samples were identical to those reported by Burk. However, position 183, not included in the classification of Burk, was a variable position in the D2 but not in the D3 variant. The majority of the D2 isolates (92.0%, 46/50) had a T (reference position) to G substitution (Fig 1) in this position, resulting in an amino acid change of isoleucine to arginine in the protein (I27R). In LCR, all positions but one (7729) agreed with the sequences found by Burk and Yamada. In this position, they found a change of A (reference position) to C in all D2 and D3 sublineages. In the present study, all D3 sublineages had the same change. However, in the majority of the D2 isolates (72.0%, 36/50), this position changed from A to T instead of A to C (28.0%, 14/50).

### Analysis of HPV16 variants by age

**Fig 2:**
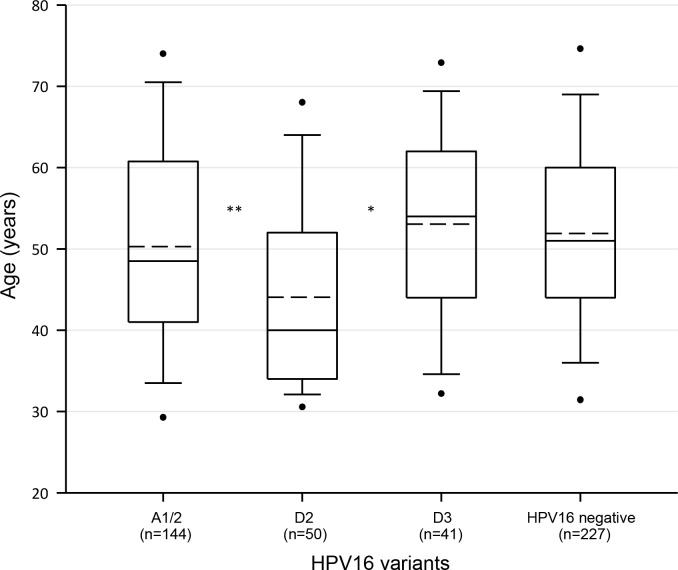
Age distribution of CC patients classified by HPV16 variants. Box plots show the age distribution of patients classified by HPV16 variant. The upper and lower boundaries of the boxes represent the 75th and 25th percentiles, respectively. The black and dotted lines within the boxes represent the median and mean values, respectively, and the whiskers represent the minimum and maximum values that lie within 1.5× the interquartile range from the end of the box. Values outside this range are represented by black circles. The statistical significance for the differences in the median age between the D2 group and the other groups was determined by the Mann-Whitney U Test. The box labeled as HPV16 negative includes samples positive for HPVs other than HPV16 (n = 220) and HPV-negative samples (n = 7). *p < 0.001, **p < 0.002.

The median age of HPV16-positive patients was 49 years (IQR = 41–60). However, large variation was observed among patients according to variants. Notably, the median age of patients positive for D2 (40, IQR = 34–52 years) was 9 and 14 years lower than that of patients positive for A1/2 (49, IQR = 41–61 years; p < 0.002, Mann-Whitney U test) and D3 (54, IQR = 44–62 years; p < 0.001, Mann-Whitney U test), respectively (Fig 2).

The percent positivity of HPV16 variants was studied in patients who were divided into two age groups: young patients (≤49 years) and older patients (≥50 years; Table 1). The percentage of A1/2 variants was relatively constant (~31%) between the two age groups, with a small non-significant decrease in the older group (Table 1). In contrast, the percentage of lineage D variants was not uniform between the groups, and the percent was inverse for D2 and D3 variants. Whereas the D2 percentage was high in young patients (15.9%) and low in old patients (5.7%), the percentage of D3 was low in young patients (6%) and higher in old patients (11.8%; p < 0.001, Pearson chi-square test; Table 1).

**Fig 3:**
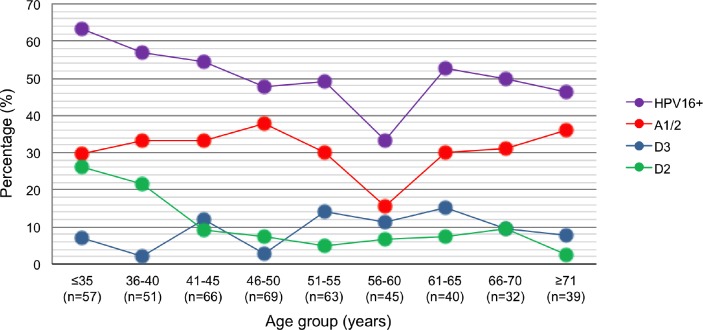
Percent positivity of HPV16 variant by 5-year age intervals. The figure shows the percentage of HPV16 infections as a whole and as segregated by lineages D (D2 and D3) and A (A1/2) based on 5-year age intervals for all CC patients (n = 462). The decrease in HPV16 and D2 percent positivity with patient age to the 51–55 year-old interval were statistically significant (p < 0.05, Spearman correlation). The HPV16+ plot has already been published in a previous paper [9], but it was included as a reference for the trend of HPV16 variants.

To investigate whether the different components of the HPV16 curve were associated with specific variants, the trends in variant percentages were analyzed by considering 5-year intervals (Fig 3). In the highest peak (≤35 years), the percentage of D variants was higher than that of A1/2 variants (33.3% vs. 29.8%), primarily because of the high percentage of D2 (26.3%). The gradual decrease in HPV16 percent positivity with patient age to the 51–55 year-old interval (r = 0.89, p < 0.05, Spearman correlation) was essentially due to the decrease in the D2 percentage (r = 0.94, p < 0.05; Spearman correlation), while the percentage of D3 and A1/2 remained relatively constant (~14 and ~28%, respectively) in these ranges. After the dip of HPV16 percent positivity in the 56–60 year-old interval, which could be simply an anecdotal finding due to small numbers, the percentage of A1/2, D2 and D3 remained relatively constant (~32%, 6% and 11%, respectively) in the latter intervals.

### Frequency of HPV16 variants according to tumor histology

As previously reported, most of the tumors analyzed were squamous cell carcinomas (SCC), with a frequency of 386 (83.5%), followed by adenocarcinomas (ACC), adenosquamous cell carcinomas (ASCC), and undifferentiated carcinomas (IND), with frequencies of 63 (13.6%), 7 (1.5%), and 6 (1.4%), respectively. The distribution of HPV16 variants was radically different between the adenocarcinoma (ACC) and squamous cell carcinoma (SCC) groups (p < 0.001, Pearson chi-square test; Totals in Table 1). The percentage of the D variants was higher than that of A1/2 variants in the ACC (36.5% vs. 19%), whereas the reverse was observed in SCC (16.6% vs. 32.9%; p < 0.001, Pearson chi-square test). This difference was more evident when the ≤ 49 year-old groups were compared (p < 0.01, Pearson chi-square test).

The median age of patients positive for different variants was similar between SCC and ACC (S2 Table). In both groups, D2-positive patients were younger than patients positive for other variants. Although the median of D2-positive patients with ACC (40, IQR: 33–49 years) was two years lower than that of patients with SSC who were positive for this variant (42, IQR: 35–55 years), the difference was not statistically significant.

### Association between HPV16 variants and early or delayed presentation of CC

We considered the older patients (≥ 50 years of age) as the reference of the outcome variable and non-HPV16 cases as the reference of the explanatory variable to investigate whether HPV16 variants are associated with early CC presentation (≤ 49 years of age). D2 increased 3.3-fold the risk of developing CC before 50 years of age (OR = 3.3, 95% CI = 1.7–6.6, p < 0.001), in relation with non-HPV16 cases. Considering SCC and ACC cases separately (Table 1), this risk was higher for ACC (OR = 6, 95% CI = 1.1–33, p < 0.05) than for SCC (OR = 2.8, 95% CI = 1.3–5.9, p < 0.01). In contrast, A1/2 and D3 were not associated with any increased risk to develop CC before 50 years of age in relation with non-HPV16 cases.

When we assessed only HPV16-positive CC cases and considered the A1/2 variant as the reference of the explanatory variable, D2 increased (OR = 2.5, 95% CI = 1.2–5, p < 0.05) and D3 decreases (OR = 0.45, 95% CI 0.2–0.9, p < 0.05) the risk to develop CC before 50 years old (Table 1). When we analyzed the risk with the opposite group as the reference (≤ 49-year group), D3 increased the risk to develop CC after 49 years old (OR = 2.2, 95% CI 1.1–4.6, p < 0.05). However, when the HPV16-positive cases were stratified by histology, the ORs were not statistically significant (S3 Table).

## Discussion

In this study, we demonstrated that HPV16 variants were associated differently between young and older women with CC. D2 was found to be associated with younger patients (≤ 49 years) and D3 with older patients (≥ 50 years), whereas A1/2 was uniformly distributed between the two age groups.

The lineage D and A genomes differ by approximately 1% [22], although for some genes such as *E2* [24], they differ by up to 2%. This difference in the genome is sufficiently large to expect functional changes between two biological entities. In fact, biological differences regarding cellular transformation have been reported between lineage D and A [25, 26, 27]. However, D2 and D3 are highly similar, differing only by a few bases. Experimental evidence can help to explain the mechanisms facilitating D2-mediated development of invasive cancer in a considerably short period. The binding of the E2 protein from lineage A variants to the four E2-binding sites within HPV LCRs diminishes the expression of E6 and E7 [28]. However, in comparative *in vitro* experiments, E2 of the D2 variant, in contrast to E2 of the A1/2 variant, did not significantly repress the transcription of the *E6* and *E7* oncogenes [29]. Furthermore, the D2 control region (LCR) is less susceptible to repression by the E2 protein based on *in vitro* experiments [30]. If this occurs *in vivo*, the expression of viral oncogenes in D2 infections can occur immediately after infection, as no E2 protein represses the viral LCR. In contrast, in lineage A infections, the progression to more advanced stages may take place more slowly, as the transcription of the *E6* and *E7* oncogenes is repressed by the E2 protein [29, 31]. Lineage A variants lose the E2 gene during viral integration into the tumor genome more frequently than D2 and D3 variants [18, 24, 29, 32]. However, this appears to occur rather late in the process of tumor development [33, 34]. Based on comparative *in vitro* experiments, the E2 protein of D3, which differs in five positions with the E2 protein of D2, represses the expression of viral oncogenes similar to the E2 protein of lineage A variants [29]. Therefore, similar to the lineage A variants, this could also explain the delay in CC progression.

Another factor that could be involved in the age presentation of the disease is the number of viral copies in the tumor [35, 36]. In almost all CCs, lineage D variants retain the *E1/E2* genes and the viral load is very high; on average, twice the load present in lineage A infected tumors [24]. As the viral load increases, the expression of viral oncogenes increases linearly [29]. However, this seems to not be a key factor in the timing of CC development, since D3 also has a high viral load. By contrast, the *E6* oncogene of lineage D variants has more immortalizing, transforming and tumorigenic abilities than *E6* of lineage A variants [25, 26, 27]. These increased abilities have been explored using the *E6* gene of D3. Since D2 and D3 share the mutations Q14H, H78Y and L83V in *E6* [22, 37], which are related to binding and degradation of p53, we assumed that D2 also has these abilities. However, it is not known whether the other change in *E6* (I27R), exclusively found in D2 and related to T cell epitopes [38], could have contributed to the differences in the risk and age of CC presentation associated with this virus in this study. This change, found in 92% of the D2-positive CCs explored in this study, has been rarely reported in other studies due to the very low occurrence of D2 [23, 39, 40, 41].

The first peak of HPV16 percentage (≤ 35-year interval) clearly results from the high frequency of D2 in those patients. Although few studies have examined the changes in HPV16 percent positivity by age in women with CC, the frequency of HPV16/18 is high in younger women throughout the world [42]. However, because D2 does not exist or is very rare outside Mexico and perhaps other Latin American countries [22], other HPV16 variants may contribute to such high frequency in most countries.

The percentages of D2 and D3 variants in this cancer series were similar to those reported in our previous study [15]. The populations analyzed in each study were different. In the first study, we analyzed women with Social Security, whereas in the present study, patients did not have any Social Security; therefore, in principle, these patients were much poorer than those in the former study. There is molecular evidence that the poorest Mexican population has a much greater Amerindian genetic component than the middle and upper classes [43]. The D lineage was not generated in America, it is too old. Recent paper on HPV16 evolution [44] suggests that a D variant ancestor was evolved before the early settlers of America crossed the Bering Strait. However, the origin of D2 and D3 variants is not completely clear, because the distribution of these variants around the world is different. Whereas the D2 distribution is by far more common in America [21, 22], D3 has a global distribution [40]. Furthermore, it is quite interesting that the population studied has a unique nucleotide at position 7729 (T) in D2, possibly representing a founder variant that has spread in Mexico. The examination of HPV16 variants in more countries may reveal the origin of these variants. To determine how the frequency of lineage D variants is related to the Amerindian genetics, it will be necessary to conduct a detailed study involving admixture mapping [45].

The D2 frequency decreased beginning at 35 years of age (26%) until 50 years of age (4%), with frequencies remaining uniformly low at older ages. This decline may be related to pre-menopause, suggesting that D2 is susceptible to hormonal influences. Inferring a causal association between hormones and HPV infection is difficult [46]. However, consensus sequences in LCR, including some associated with hormonal response, have several mutated sites in lineage D variants. One of them (A7729T), located in a putative glucocorticoid response element (GRE) binding site [47, 48, 49], is detected mostly in the LCR of D2. Whether this change could be involved in hormonal response and with the shorter development of CC is not known.

Finally, it can be stated that one of the strengths of this study was the large number of HPV16-associated CC cases positive for lineage D that were analyzed, especially those positive for D2. Considering this, in this case-case study, we have statistically proved the higher risk conferred by D2 for the development of CC before 50 years of age, both in SCC and ACC, as compared to the older-aged group. However, to compare the risk that these HPV16 variants confer for the development of the disease between infected and uninfected women of different ages, it is necessary to conduct a case-control study. Since the incidence of D2 and D3 infections in healthy women is very uncommon, a large control group is required to make a robust analysis considering age stratification.

## Supporting Information

S1 Table Distribution of HPV16 variants in cervical cancers positive for single HPV16 or HPV16 plus another HPV type (n = 235). (XLSX)

S2 Table Median age of HPV16-positive patients classified by variant type and tumor histology. (XLSX)

S3 Table Association between D variants and early presentation of HPV16-positive cervical cancer cases stratified by histological type. (XLSX)

## Abbreviations

CC: cervical cancer
HPV: human papillomavirus
AA: Asian-American
E: European
SCC: squamous cell carcinoma
ACC: adenocarcinoma
FIGO: International Federation of Gynecology and Obstetrics
